# Self-Care Practices and Their Role in the Control of Diabetes: A Narrative Review

**DOI:** 10.7759/cureus.41409

**Published:** 2023-07-05

**Authors:** Farhan Ahmad, Shiv H Joshi

**Affiliations:** 1 Department of Community Medicine, Jawaharlal Nehru Medical College, Datta Meghe Institute of Higher Education and Research, Wardha, IND

**Keywords:** diabetes mellitus, self-care practices, complications, blood glucose level, risk factors, disabilities

## Abstract

Diabetes mellitus (DM) is a long-standing, continuously growing metabolic ailment in which levels of glucose in the blood increase due to a total (DM of type 1) or incomplete (DM of type 2) decrease in the level of the hormone insulin. Diabetes mellitus affects a large number of individuals worldwide, and as more people develop the disease, the burden will double from what it is now. The requirements of people suffering from diabetes are not only confined to the control of blood glucose; there is also a need to prevent disabilities, side effects, and difficulties in rehabilitation. Studies suggest that seven self-care practices for individuals suffering from this disease have shown good outcomes. Those practices include assessment of sugar levels in the blood, consuming healthy foods, remaining physically active, taking medications regularly and on time, maintaining healthy behavior, and decreasing risk factors. All of these practices collectively have shown good results in maintaining blood glucose levels, decreasing side effects, and increasing life expectancy in people with diabetes mellitus. Those who have DM and practice self-care have shown positive results by reducing the complications of DM, decreasing its progression, and leading to a huge reduction in the burden due to DM. Despite these positive changes, people sticking to these self-care practices are very few, specifically when we see broad and chronic changes. There are many positive contributing factors, such as social factors, demographic factors, and various socio-economic factors, but the role of physicians in increasing the practices associated with personal care for people with this disease is crucial and most important for the desired outcome. Keeping in mind the burden and multidimensional nature of the disorder, proper systematic and combined efforts are needed to increase these self-care practices in patients with diabetes to reduce any chronic side effects and complications.

## Introduction and background

Diabetes is a chronic disease that occurs either when the pancreas does not produce enough insulin or when the body cannot effectively use the insulin it produces. Diabetes has its effects all over the body's system due to the derangement of metabolical activities produced due to an increase in blood glucose level, specifically when adequate maintenance of diabetes during a certain period is below the optimal level [1]. Previously, it was thought that diabetes happens in just developed nations, but newer studies and results have shown that there is an increase in newer patients with type 2 diabetes, with commencement being very early and mostly associated with side effects and complications in developing nations. Mostly, the complications that are associated with diabetes are neuropathy, cardiovascular diseases, retinopathy, and nephropathy. These complications are responsible for disabilities and casualties among people suffering from diabetes. According to a report by the WHO, diabetes mellitus (DM) affects approximately 346 million people worldwide, and if effective action is not taken to treat the condition, this number is predicted to double by the year 2030. About 80% of casualties reported due to diabetes occur in middle- and low-income nations [2]. India leads the list globally with around 3.2 crore cases of this disease, and this figure is likely to go to 7.94 crore by the end of 2030. The majority of the surveys have shown 10-16% of the population from urban areas and 5-8% of the population from rural areas are affected by diabetes in India, as well as Sri Lanka [2,3].

Objective

The main aim of the article is to underline the importance of self-care practices and their role in controlling diabetes mellitus and preventing the complications associated with it. Self-care practices enable the maintenance of optimal glycemic control for patients with diabetes through a comprehensive lifestyle, medication adherence, and monitoring of blood glucose levels, preventing the complications of diabetes. The goal is to address and deal with the requirements and desires of individuals struggling with diabetes through these self-care practices, which lead to a better quality of life. Timely follow-up of patients with diabetes is of the utmost importance to avoid complications. It is important that patients understand the benefits of self-care practices and their role in preventing the complications of diabetes.

Methodology

**Figure 1:**
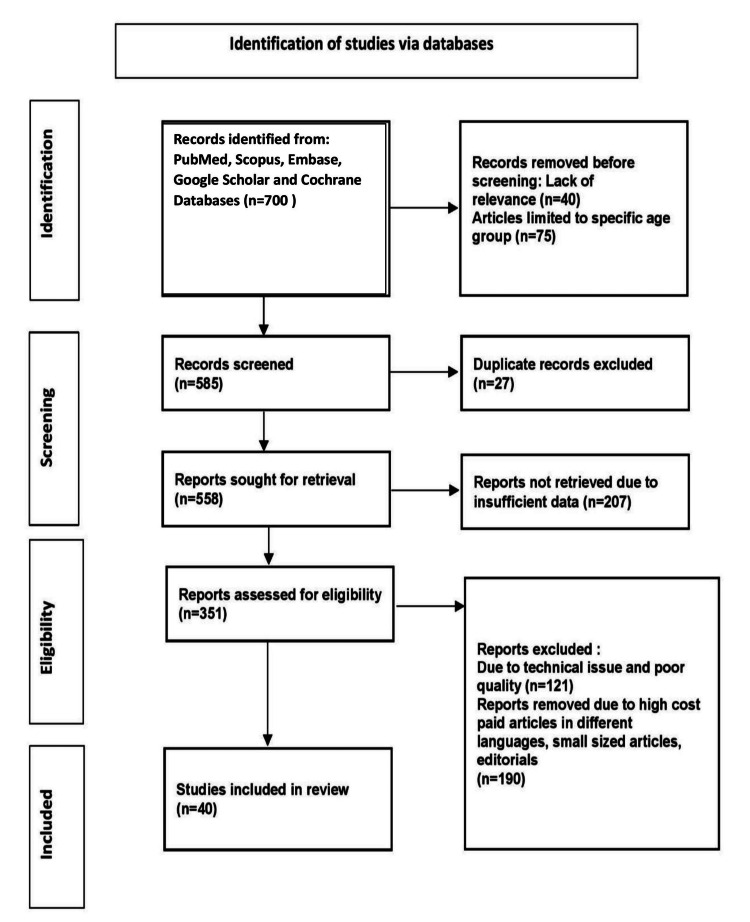
Research methodology by PRISMA method PRISMA: Preferred Reporting Items for Systemic Reviews and Meta-Analysis

The following keywords were used to search in PubMed, Scopus, Embase, Google Scholar, and Cochrane databases: diabetes mellitus, diabetes self-care practices, diabetes management, personal care in diabetes, and treatment modalities of diabetes. The search yielded 700 articles, of which 40 research articles were selected for research. The methodology of the Preferred Reporting Items for Systemic Reviews and Meta-Analysis (PRISMA) method is shown in Figure 1.

## Review

Self and personal care in diabetes

The definition of self and personal care in this disease has been defined as a revolutionary change in the progress of education or being apprised by studying to remain alive with diabetes. Since most daycare activities for diabetes are managed by the families of the patients or by the patients themselves, there is an urgent need for measures to be taken to self-control diabetes. Six self- and personal-care activities and practices among individuals suffering from diabetes have shown positive outcomes (Table 1). They are monitoring blood glucose, eating a healthy diet, remaining physically active, remaining adherent to the treatment regime, taking medications on time, and reducing risk behaviors. These practices can be beneficial for physicians and educators dealing with diabetic patients as well as for researchers introducing newer measures for diabetic care and control [4]. These self-care practices are habits developed by individuals at risk or suffering from diabetes so that the disease can be managed by themselves without much help or intervention. The six practices mentioned above have shown good outcomes in controlling blood glucose levels, reducing complications of diabetes, and maintaining and improving the quality of life. Self-care associated with diabetes requires the patient to undergo lifestyle, eating, and dietary changes with the help of physicians, which leads to a successful change in behavior and attitude with self-confidence toward dealing with diabetes [5].

**Table 1:** Self-care practices and their outcome on diabetes and its complications

Self Care Practices	Outcome
Monitoring blood glucose	Shown positive outcomes in control of diabetes and complications associated with it
Eating healthy diet	
Physical exercise	
Adherence to the treatment regimen	
Taking medications regularly and on time	
Reducing risk behaviours	

Importance of self and personal care in the treatment of diabetes

Training associated with personal care and self-management of diabetes begins with the proper knowledge of instructions, which are based on the needs of the patient. A trainer or instructor that deals with diabetes makes the patient arrange, differentiate, and move ahead in the direction towards the objectives that lead to changes that will give self-confidence, lead to a better quality of life, and manage any complication. Since diabetes is a multidimensional disorder, physicians should not single-handedly give discrete measures of data and guidance [6-9].

Tips for personal care and self-management in diabetes mellitus

Self-care practices are a set of behavioral practices used by individuals suffering from diabetes in order to manage and control the disease on their own. These self-care practices are found to have an association with blood glucose levels and thereby reduce the incidence of complications associated with diabetes. Various studies and evidence show that when a patient performs self-care practices in a correct and systematic manner, they can improve blood sugar control. These practices have proven effective in achieving the therapeutic goals of diabetes [7].

The following tips for personal care, i.e., daily exercise, diet, quitting smoking, foot care, fiber intake, tooth care, eye care, and stress management, will help in the self-management of diabetes. The details of each are given below.

Daily Exercise 

Physical exercise is of the utmost importance in dealing with diabetes. Daily exercise aids in the digestion of food. Daily exercise helps to control the level of blood sugar in diabetic patients [7].

Diet

Blood sugar levels can be maintained by avoiding foods with high-calorie counts, cutting back on salt and sugar, and avoiding junk food [8].

Quit Smoking

Individuals diagnosed with diabetes mellitus should quit smoking and remain completely away from drugs because they will cause blood vessels to narrow, which will further cause a decrease in blood circulation [9].

Foot Care 

Individuals suffering from DM type 2 should properly wash and clean their feet using warm water, then dry and clean them neatly. Any edema or injury to the foot has to be given the utmost observation, and consultation from a physician should be sought [10].

Intake of Fiber

Food with a high fiber content should be consumed. High fiber intake enhances the process of digestion, maintains the blood sugar level, and decreases cholesterol [11].

Tooth Care

After every meal, proper brushing and flossing of the teeth will prevent infection of the gums. Inflammation of the gums, indicating redness and swollen gums, needs immediate intervention [12].

Eye Care

Regular eye checks need to be done, failure of which will lead to retinopathy. Regular checkups can prevent this complication [12].

Stress Management 

Management of stress with methods like yoga should be implemented and managed effectively because the hormones produced in response to stress lead to improper functioning of insulin, leading to an increase in blood glucose [8].

Self-care practices 

Self-care involves intentional methods to take care of physical, emotional, and mental health. Any patient with a chronic illness may experience effects on their general health and way of life from changes in their choices and behaviors, which is the best part of personal self-care. These practices include different things that need to be taken care of, such as medicines, exercise, food, sleep, emotions, medical facilities, and care. The American Diabetes Association (ADA) mentions that keeping an eye on the intake of carbohydrates and fiber, undergoing weight loss, and cutting short the intake of cholesterol, saturated fat, trans fat, and salt are the basic things that need to be done for the treatment of this disease. Along with this, patients also require additional practices to review individual and social things for the betterment of the treatment of diabetes mellitus [13-15].

Obesity is one of the major issues for individuals suffering from diabetes mellitus type 2. Dietary changes are the principal factor in dealing with diabetes mellitus since they can lead to weight loss and ultimately help in the management of obesity, which further helps in the intervention of diabetes mellitus. There are many fewer studies done that have primarily focused on the management of obesity in children [16]. Also, a limited number of studies have paid attention to the treatment of adolescent patients suffering from type 2 diabetes mellitus, which has included dietary control along with exercise and attitude changes. The results from these studies conclude poor and pessimistic results with respect to the influence of food on managing the end result, but independent results of diet modification were not counted. A recent study analyzing a long-term diet with reduced glycemic burden and a standardized diet with less fat in adult individuals with obesity has shown that a diet with less fat can be a better replacement for a conventional diet with less fat for decreasing the long-term side effects of diabetes in obese patients with diabetes mellitus type 2 [17,18].

Personal care with physical exercise is an internal part of managing this disease and helping with the motion of skeletal muscles [19]. The goal of physical exercise is to achieve blood sugar level regulation, improve the action of insulin, improve the metabolism of protein and fat, avoid complications of diabetes, and increase life quality and expectancy. Sufficient physical exercise leads to lower levels of HbA1c. The only condition included is that it should be integrated with dietary advice. Younger generations should know the needs and significance of regular physical exercise, which helps them lose calories, reduce weight, and maintain blood glucose levels. In addition, the combined changes in diet and daily physical activity aid in balancing normal weight and increasing weight loss in individuals with obesity (Table 2) [20,21].

**Table 2:** Activities associated with diabetes self-care practices

Category	Activities
General	Reducing the risks associated with diabetes complications.
Meal and nutrition	Ensure sufficient intake of essential vitamins, minerals, proteins, and fiber through meals.
Physical activities and exercise	Exercise is one of the most important self-care practices for diabetes management.

Self-care management education for diabetes

Although genetics is responsible for the development of this disease, twins with a monozygotic constitution have shown that environmental influences also play a crucial role in the progress and development of this disease. People with this disease have shown positive effects and outcomes on their development and progression of diabetes by engaging in self-care activities [22,23]. This active participation can have a positive impact only if patients suffering from this disease and their physicians are brought to notice about undertaking effective self-care and personal care for diabetes. It is understood that people who have more information about the disease will have a better understanding of the disease, which will have positive effects on disease development and progression as well as complications arising out of it [24].

The Association of Endocrinologists (clinical) in America depicts the significance and need for patients to remain physically active and have some sort of knowledge of their self-care activities. The Association of American Diabetes has done studies and reviewed the results of standards for diabetes self-care and personal management and understands that there is a four-fold increase in complications of diabetes in people who have not taken any advice or education related to self-care practices. A study of self-management education for individuals dealing with type 2 diabetes has shown that there is improvement in blood glucose control in the follow-up. However, this improvement in blood glucose decreases after one to three months of stoppage of self-care activities, which suggests that education on self-management is helpful in reducing glycosylated hemoglobin [25-29].

Education related to diabetes is crucial, but it must be inculcated in life or through self-care activities for the betterment of individuals. Self-care means activities related to diet control, increasing physical exercise, ignoring foods high in fat, monitoring blood glucose, and taking care of your feet. Reducing the levels of glycosylated hemoglobin might be the eventual target of self-management, but it can’t remain the only target in the individual's care. Certain changes in personal care should be reviewed for progress and the development of a behavior change [30].

Monitoring glucose levels in the blood of patients with diabetes is an important cornerstone of diabetes care and can help patients participate in achieving glycemic targets. The ultimate aim of monitoring blood glucose is the overall assessment of glucose control and taking optimal steps in time to achieve an optimal blood glucose level. Monitoring glucose levels in the blood gives information about current blood glucose levels, allowing individuals to assess their progress and undergo adjustments in medication, diet, and physical exercise so that optimal blood glucose levels can be achieved [31]. A recent survey has shown that regular physical exercise has a positive impact on improved health outcomes in diabetics, irrespective of their weight loss. The American College of Sports Medicine and the National Institute of Health suggest that all adults, including those suffering from this disease, should perform daily physical exercise [32-35].

Compliance with activities related to self-care

Adherence to the treatment regimen for diabetes is an important area of interest and worry for physicians and researchers, although many studies and research have been done previously in this regard. In diabetes, individuals need to adapt to a plan and follow it strictly. This plan should comprise many behavioral changes and actions to take care of diabetes on a regular, day-to-day basis. All these changes and actions should comprise positive lifestyle changes, which include dietary planning and regular physical exercise; taking proper medications daily, which comprise insulin or oral medications that act as hypoglycemic agents as and when indicated; keeping an eye on blood sugar levels; managing symptoms related to diabetes mellitus; undertaking guidelines related to foot care; and taking care of diabetes or other problems related to health. The regimens that are already there for diabetes are complicated further since there is a need to add and integrate all these behavioral aspects into the patient's day-to-day life [36].

The maximum number of patients can effectively decrease the long-term complications arising from diabetes by improving self-care activities. But still, even if the advantage of these activities is life-saving, adherence to these self-care activities is found to be very low. In a study done and conducted on individuals with diabetes, the results showed that only 30% of individuals were adherent to the treatment regimen, and non-adherence was higher, especially among groups with lower socio-economic status. On the other hand, one part of the truth is that only sticking to type 2 diabetes self-care and personal care activities will not lead to good control of blood sugar levels. Various research studies done globally show that control of metabolism is a combination of multiple things and not just individual adherence to self-care and personal care activities and treatment regimens. In a trial done in America, it was noticed that individuals are more likely to grasp and make changes when each change is implemented individually. Positive outcomes may therefore vary, and it depends on the changes that are made to enact them, whether singly or simultaneously [37,38].

Barriers associated with self-care of diabetes

The importance of physicians and healthcare workers in the care of patients with diabetes is widely known and recognized. Various cultural barriers, such as higher costs and finances, the satisfaction of patients with medical care, much less and poorer access to diabetic drugs, doctor-patient relations, symptoms with varied degrees, an improper balance of health care providers between rural and urban regions, and socio-demographic barriers, have varied restrictions on self-care and personal care activities in progressing countries. In a survey done regarding barriers from the physician's point of view in regards to care of diabetes, various aspects like expenses and affordability by the individuals, belief and willpower from physicians that medication and treatment regimens cannot treat patients completely, and zero confidence in themselves regarding their ability to change the patient's attitude and behavior came forward. Another such study was done that focused on both patient- and physician-related factors [39]. Patient-related factors comprise social support, financial assistance behavior, attitude, education and knowledge regarding diabetes, language, and cultural capabilities, while physician-related factors include self-beliefs, knowledge regarding diabetes, attitude, and communication factors [40].

## Conclusions

To reduce the burden of morbidity and mortality associated with diabetes, there is an utmost need for self-care activities in more than one domain, which also include physical exercise and activities, diet control, taking proper medications and their adherence, and monitoring blood glucose levels in the patients. Although many other multiple factors, such as social support, demographics, and socio-economic elements, can be seen as positive contributors to promoting self-care and personal care activities related to individuals with diabetes.

Also, the role and responsibility of physicians in facilitating and promoting self-care are important. Since it is understood that diabetes is a multidimensional problem, a systematic, integrated approach is needed to facilitate self-care activities among individuals suffering from diabetes to avoid and prevent long-term chronic complications. Along with these things, it also requires patient self-capability and different self-managing abilities and techniques. Diabetes is a life-long disease, and the need to undergo changes in lifestyle, self-care, and management is identified as the principal thing in the treatment of diabetes mellitus.
